# Legacy of culture heritage building revitalization: place attachment and culture identity

**DOI:** 10.3389/fpsyg.2023.1314223

**Published:** 2024-01-23

**Authors:** Suk Ha Grace Chan, Wing Han Helen Lee, Binglin Martin Tang, Ziyi Chen

**Affiliations:** ^1^School of Liberal Arts, Macau University of Science and Technology, Macau, Macao SAR, China; ^2^Institute of Management and Health, University of Wales Trinity Saint David, Swansea, United Kingdom; ^3^Faculty of International Tourism and Management, City University of Macau, Macau, Macao SAR, China; ^4^Faculty of Innovation and Design, City University of Macau, Macau, Macao SAR, China

**Keywords:** cultural identity, heritage building, place attachment, post-experience behavior, revitalization

## Abstract

**Introduction:**

Cultural heritage buildings are revitalized to promote culture instead of being neglected or demolished. For locals, the revitalization of heritage buildings symbolizes a commitment to the community and a taste of collective memory. The study attempts to test the effectiveness of heritage cultural building on visitors’ post-experience behavior through cultural identity and place attachment. “Cultural activities engagement” and “knowledge transfer” serves as moderating attributes. Their moderating the effects on cultural identity and place attachment are examined, respectively.

**Method:**

A valid sample size of 348 from four heritage buildings located in Hong Kong: Tai Kwun, Police Married Quarters (PMQ), Mei Ho House and The Mills. A data analysis platform for PLSSEM is chosen for this study.

**Results:**

Results demonstrate that the effectiveness of heritage building revitalization on visitors’ local cultural identity and emotions leading to place attachment.

**Discussion:**

In our study, the effectiveness of heritage building encompasses three elements which includes appearance and components; technology and planning as well as contribution. Knowledge transfer positively moderates the effectiveness of heritage building revitalization and cultural identity. However, cultural activity engagement negatively moderates place attachment. Findings also exhibit that place attachment in heritage building revitalization leads to a positive experience extension. For the practical contribution, the study provides insights to policy makers and planners for historic building design such as appearance and components, technology in facilitating local visitors’ local identity.

## 1 Background

The study scrutinizes the effectiveness of vitalized heritage building on local visitors’ post-experience behavior through local cultural identity and place attachment. The historic buildings selected for the study are located in Hong Kong–Tai Kwun, Police Married Quarters, Mei Ho House and The Mills. The study primarily encompasses three major parts. The first part of the study explores the relationship between the effectiveness of the vitalized heritage buildings and local cultural identity. The second part examines the relationship between the local cultural identity and place attachment. It is posited that cultural activities engagement have a positive impact on place attachment. The last part of the study emphasizes the relationship between the place attachment and local visitors’ post-experience behavior. In this study, the post-experience behavior takes two forms – experience intensification and experience extension. This study examines the moderating effects of knowledge transfer and cultural engagement on visitors’ post-experience behavior. Upon literature review, six hypotheses are developed to frame the study. The study integrates Attachment Theory and Social Identity theory for investigation. For the methodology, the study adopts quantitative approach through survey for data collection. Findings and discussions are laid and the study ends with implications, limitations and future research.

Revitalizing historic buildings aims to maintain a standard that allows visitors to enjoy the cultural resource and create a sense of belonging in the community (Zhang and Smith, 2019; Wang, 2023). The sustainability goals extend beyond protecting the buildings, as they can create value within the local community and influence future behavior (Idrus et al., 2010). Several international examples have proven the value of heritage building revitalization, including Germany’s Zollverein Park, Japan’s Itonowa in the Shimabara district of Kyoto Shiroishi city, Singapore’s ChinaTown and open-air museums in the United Kingdom (Lam et al., 2022). The proven record remarks the success of urban regeneration and the needs for heritage revitalization. More importantly, it serves as benchmarks for rejuvenating heritage buildings and facilitating local visitors’ behavioral intention in Hong Kong.

**FIGURE 1:**
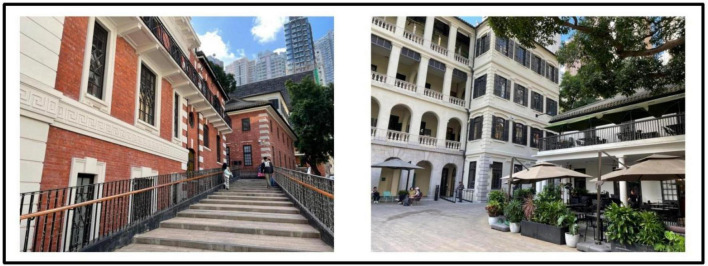
Exterior and Interior from Tai Kwun. Author’s Self-Photography.

**FIGURE 2:**
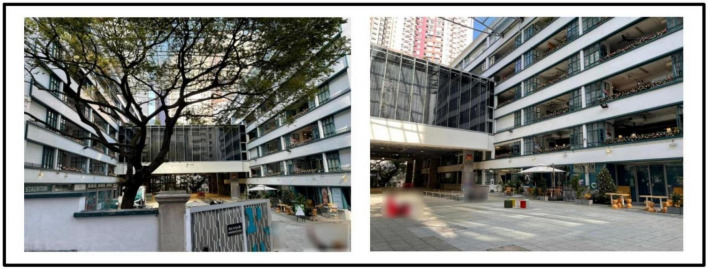
Exterior and Interior from Police Married Quarters (PMQ). Author’s Self-Photography.

**FIGURE 3:**
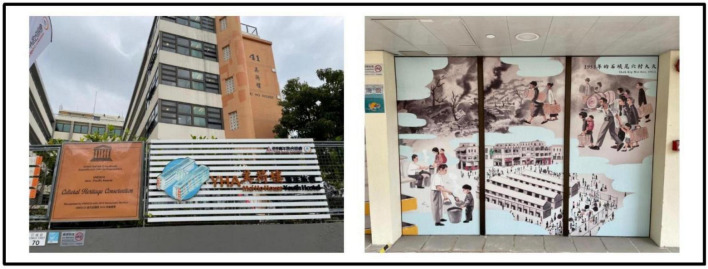
Exterior and Interior from Mei Ho House, a youth hotel and museum. Author’s Self-Photography.

**FIGURE 4:**
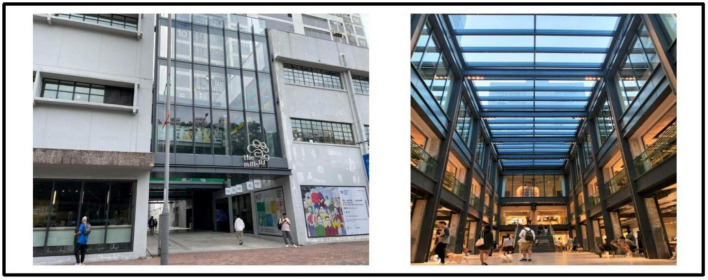
Exterior and Interior from the Mills. Author’s Self-Photography.

In Hong Kong there are numerous historical buildings featuring its cultural nostalgia (Chan and Lee, 2017). The project made through the Partnership Scheme (Ho and Hou, 2018) and aims of the project was to conserve the historic building for cultural landmark and to improve the life of near-by residents (Conserve and Revitalise Hong Kong Heritage, 2023). Four buildings in urban areas are involved for revitalization includes Tai Kwun, a central hub for heritage arts (formerly Central Police Station Compound, see Figure 1), PMQ, a creative industries landmark (Central School; formerly Hollywood Road Police Married Quarters, see Figure 2), Mei Ho House, a youth hotel and museum (formerly Public Housing, see Figure 3) and The Mills, a center for heritage and shop floor (formerly Nan Fung Cotton Mills, see Figure 4; Lam et al., 2022). Barber (2020) realized that importance of cultural values of the historic building brought to the community. Liu et al. (2022) highlighted the importance of cultural values brought to the community and facilitated cultural tourism. Besides, limited research on the above four revitalized historic buildings together is found and target local visitors’ for as respondent. In view of the above, the study attempts to fill the above gaps to examine its impact of the four revitalized historic buildings on local visitors’ post-experience behavior.

Cultural heritage revitalization such as historic monuments facilitates urban regeneration and bring about socio-economic values to the community and its neighborhood (Douglas, 2005; Ashworth, 2011; Douglas-Jones et al., 2016). However, these positive values are not always explicit to the locals and visitors. Heritage buildings can also contribute to environmental sustainability and education for future generations (Kincaid, 2000; Wilkinson et al., 2009; Spina, 2019; Ali and Qi, 2020). Chan and Lee (2008) highlighted the historical values of heritage based on six benefits, including historical, architectural, group, social and cultural, authenticity and rarity values (Kee, 2019). Additionally, relevant knowledge and engagement in cultural activities can influence visitors’ behavior and their post-experience behavior. Limited studies have focused on the visitors’ viewpoint regarding revitalizing historic buildings and highlight the breadth of perspectives in environment to individuals place attachment and social identity research (Yung and Chan, 2011; Villanueva et al., 2017; Barber, 2020). Implementation of knowledge transfer and cultural activities engagement may play a crucial role in affecting local visitors’ perception of a destination, which has been lacking in previous studies. This work aims to answer the following questions: To what extent does historic building revitalization stimulate local cultural identity? Do local cultural and placement facilitate post-experience behavior?. Do “knowledge transfer” and “cultural activities engagement” moderate local visitors’ post experience behavior? This study incorporates attachment theory and social identity theory as the core of the conceptual models to examine the effectiveness of heritage building revitalization on visitors’ post-experience behavior with knowledge transfer and cultural activities engagement as moderating attributes. The study objectives include:

• to examine the influence of revitalizing heritage buildings on cultural identity;
• to investigate the relationship between local cultural identity, place attachment and visitors’ post-experience behavior; and
• to investigate the moderating effect(s) of “knowledge transfer” “cultural activities engagement” on visitors’ post-experience behavior.

This research implies theoretical and practical contributions. For the theoretical contributions, Firstly, it expands the existing literature by exploring the impact of the effectiveness of revitalizing historic buildings on local visitors’ post-experience behavior from visitors’ perspective. Previous studies emphasize the relationship between the historic building and heritage revitalization. To go beyond the scope, the study explores the impact of historic building on post-experience behavior. It helps enhance diversity of research with different spectrums, Secondly, it integrates attachment theory of environmental psychology and social identity theory of social psychology to contextualize the impact of historic building vitalization on local community and visitors’ behavioral intention. Thirdly, it highlights the special values of revitalizing historic buildings for the community and their contribution to sustainable behavior.

For practical contributions, firstly, the study provides insights to destination marketers and planners. Secondly, revitalization of historic building does not only help sustain the cultural heritage, but also facilitates visitors’ attachment to the place. More importantly, the enhanced level of place attachment leads to a positive behavioral intention. The policy makers, destination planner and marketers may reconsider the urban building redesign. Ultimately, the study takes a unique approach by applying these theories to a destination context, providing valuable contributions to academia and practical applications.

## 2 Literature review

### 2.1 Attachment theory and social identity theory

In the study of environmental psychology, scholars often adopt attachment theory and place identity concepts in their literature review (Tsai, 2016; Loureiro et al., 2022). Place attachment is used by psychologists to investigate how individuals feel about a particular place. Based on early attachment theory, which highlights a person’s sense of balance between closeness to and distance from key people in their life (Ainsworth, 1967), individuals’ attachment experiences influence their behavior and interactions with others. Place attachment refers to individuals’ emotional ties to their home, communities and societies, particularly their attachment to local environments. The emotional connection individuals feel toward a place may reflect their sense of belonging and connection (Tsai, 2016). Buildings that preserve common memories can become symbols of local cultural identity for residents (Rugwiji, 2019; Cittati et al., 2022). Therefore, revitalized architecture has the potential to reinforce cultural identity differences between local residents and other communities, stimulating the development of local attachments.

The definition of place attachment associate with individuals and their response to place. The psychology of place for individuals which need a positive environment in which to live (Scannell and Gifford, 2017). Others defines the attachment studies which based on person, place and process which affect the individual behaviors (Scannell and Gifford, 2010). Place attachment benefits to individuals’ emotional ties to their home, communities and societies, particularly their attachment to local environments. The emotional connection individuals feel toward a place may reflect their sense of belonging and connection (Tsai, 2016). The attachment common understand “place” as a location object of mental bonds which can provide attributes of physical feature and time where they spent in an environment (Diener and Hagen, 2022). The synergies between place identify for a place sense of place and place identity in provide psychological interaction that occur a crucial aspect to the environment (Scannell and Gifford, 2010). The place belongingness stimulates emotional approach with corned the place “prefer this place” for connection in environment that satisfies individual needs (Tuan, 1974). Furthermore, individuals will take pride to the environments and sense of wellbeing (Scannell and Gifford, 2010), which benefit engagement activities include post-environmental behavior (Anton and Lawrence, 2014; Daryanto and Song, 2021).

Loureiro et al. (2022) emphasize that visitors who engage in outdoor activities enhance pro-environmental behaviors. Promoting education to visitors and protecting heritage buildings can be achieved through outdoor activities. Proshansky (1978) suggests the importance of the physical environment in the development of self-identity. Identity is formed through the relationship individuals establish and the nature of the interactions that take place in a particular environment (Bernardo and Palma-Oliveira, 2012, 2013). The effectiveness of heritage building revitalization strongly reflects local histories and past cultural backgrounds, evoking pleasant memories for local visitors. Therefore, successful heritage building revitalization can create a memorable experience, facilitate a clear understanding of a place and provide educational opportunities for future generations with a focus on sustainability. Revitalized heritage buildings, with effective knowledge transfer to visitors and high engagement in cultural activities, can stimulate emotional ties, enhance visitors’ enjoyment and foster a sense of place attachment. Cultural activities can aid visitors in understanding the history of the heritage building, gaining knowledge and enhancing their collective memory and differentiate themselves from the others. Therefore, culture engagement activities affect their feeling with environmental setting comprised the perception of the environment and provide with their own identity to their place.

Previous study argued that place attachment have a relationship with social identity since the social identification with their place where people think that they can bring the well –being from the environment (Maricchiolo et al., 2021). One of the main reasons is they serves themselves as part of the group or local social group or social identity affect their place attachment. Social identity well defines as people build a perception of themselves by means of abstract social categories and their perceptions become part of people’s self-concepts (Sim et al., 2014; Carter and Bruene, 2019). Social identity influences individuals’ motivation to engage in behaviors that serve the group, as it creates a sense of self-worth and reinforces membership status (Li et al., 2022). However, the inclination of individuals to engage in these behaviors can be amplified by their prosocial motivation. From a motivated information processing perspective, prosocial motivation stimulates the search for information and the processing of group-level attributes that contribute to collective success (Super et al., 2016).

Social identity theory suggests that individuals perceive group members as part of themselves and define themselves as part of a larger whole (Stets and Burke, 2000; Riketta, 2005; Spears, 2011). The theory posits that people categorize the world into “them” and “us,” and they consciously or unconsciously reinforce the differences between groups (Tajfel et al., 1979). As a result, individuals from different cultures have relatively independent notions of social identity. People with similar social identities can cooperate and communicate more effectively because they share attachments to the same group (Smaldino, 2019). Conversely, Scholars (Lewicka, 2011; Ramkissoon et al., 2011) argue that individuals with low identity tend to exhibit low levels of attachment to both groups and places, contributing to their sense of isolation.

Culture is considered a powerful tool for distinguishing groups (Da’as and Zibenberg, 2021). Buildings that preserve common memories can become symbols of local cultural identity for residents. Therefore, revitalized architecture has the potential to reinforce cultural identity differences between local residents and other communities, stimulating the development of local attachments. Buildings that preserve common memories can become symbols of local cultural identity for residents (Rugwiji, 2019; Cittati et al., 2022). Heritage building revitalized has potential to reinforce cultural identity between local residents and other communities, stimulating the development of local attachments. Under this reason, heritage building revitalization provide culture engagement activities can shape individuals behavior.

According to Vaske and Kobrin (2001) identify the relationship between the place attachment and result may lead to sustainable place behavior which lead to environmentally-responsible behavior in other aspect of visitors’ life. Therefore, evoke place attachment can encourage visitors to make protection to a place (Halpenny, 2010). Scholars also confirmed that in addition to the emotional connection, gain knowledge about a place, increases the likelihood that individuals will demonstrate place-protective behaviors (Walker and Chapman, 2003). It is logic to argue local visitors with place attachment to the heritage building revitalization where they are willingness to protect the place since they feel attached. The more willing to protect the heritage building, more willing to attend the culture activities engagement. As result, gain individuals post-experience behavior in this place (Alawadi, 2017; Li et al., 2021). Visitors are more willing to present the experience and sharing to the others (Arnould and Price, 1993). Under the foundation of the place attachment and culture activities engagement, as result they may stimulate the post –experience behavior.

### 2.2 Linking heritage building revitalization, place attachment and cultural identity

Revitalization entails giving new life, vitality and strength to an area (Lam et al., 2022). The revitalization of heritage buildings can be achieved by either maintaining the building’s original appearance or introducing new elements to meet new requirements (Nowogońska, 2019).

Local cultural identity refers to a sense of belonging to a particular cultural or ethnic group that develops through membership in a specific culture, involving the learning and acceptance of traditional cultural and social structures (Lustig et al., 2006). Cultural identity is regarded as an important perspective for exploring the development mechanism of cultural heritage and as a factor influencing cultural heritage tourism (Wang and Hu, 2014). Individuals with a strong local cultural identity contribute to the protection, development and retention of local areas (Pretty et al., 2003).

According to attachment theory, attachment can be defined as an emotional connection with a person or object that is linked to a person’s sense of security (Chen et al., 2014). Place attachment refers to the positive association or connection between a person and a particular place. Over time, human mental activity, emotional expression and mental health are positively associated with local attachment. A significant manifestation of this association is that individuals feel a sense of closeness to specific places (Aliakbarzadeh Arani et al., 2022).

Building revitalization can improve the efficiency of local resource utilization and reduce pressure on local ecosystems (Andrić et al., 2017). The revitalization of the landmark barn in Mas di Sabe, Italy, has strengthened the cultural identity of the local people and better preserved the collective memory of the area (Cittati et al., 2022). Similarly, the revitalization of heritage buildings in industrial areas in Xi’an, China, demonstrates that buildings once used for industrial purposes can be transformed into liveable and environmentally friendly residences that are popular with locals. These revitalized industrial heritage buildings are expected to foster a strong attachment among younger generations to their hometowns (Li et al., 2015). In a study by Lee and Jeong (2021), satisfactory visitation is noted as an important prerequisite for developing place attachment.

The preservation of historical heritage buildings and the promotion of social connections, place attachment, social cohesion and community identity have significant benefits (Yung and Chan, 2012). Heritage building revitalization provides significant contributions to the local economy and community planning. Overseas examples have demonstrated that after building revitalization, there is an increase in business opportunities for entertainment, attracting non-local visitors who generate economic activities. Value enhancement is not limited to a quantitative economic approach; it also encompasses the connection with local identity. Yung and Chan (2012) argue that local sustainability behavior is influenced by built heritage projects, where locals can enhance their sense of cultural identity through the atmosphere of the place and the quality of the environment. Locals play a crucial role as a motivational force behind the design for the conservation of built heritage, establishing a link to community cultural identity (Tweed and Sutherland, 2007). The revitalization of heritage buildings can increase locals’ pride and understanding of the culture within their community, positively affecting their sense of belonging (Pignataro and Rizzo, 1997).

Study (Halpenny, 2010) confirmed residents have place attachment, they are willing to engage in the local social activities. There is a relationship between place attachment and post –experience behavior. Bricker and Kerstetter (2000) identified that whereas place-dependent recreationists were generally more concerned with resource development and maintenance, recreationists displaying high tendencies toward place identity were more often associated with an interest in resource preservation and maintaining primitive settings. Other studies found a strong positive correlation between rural residents level of support for conservation planning and their level of attachment to the landscape which depends on the different social outdoor activities (Vaske and Kobrin, 2001; Buta et al., 2014). Visitors are more prefer to visit and expand their post-experiences behavior. Therefore, the preservation of historical building can create certain value for local community. The three major elements in evaluating heritage revitalization projects include building appearance and components, planning techniques and public acceptance (Yung and Chan, 2012). Therefore, it is reasonable to argue that the effectiveness of heritage building revitalization can lead to local acceptance and foster post-experience behavior.

### 2.3 Effectiveness of heritage building revitalization and local cultural identity

Architecture and culture contribute to a sense of place, social traditions and cultural identity spanning centuries of history (Hassler et al., 2002). Although architectural heritage is rarely studied in relation to cultural identity, previous research has highlighted that local residents, tourists, and experts agree that making a region unique with its cultural identity requires a focus on maintaining or even revitalizing heritage buildings. There is a strong link between resident dependence and the renewal of heritage tourism, suggesting that resident dependence increases support for tourism development (Chan and Chen, 2010). Study highlighted in the urban identity which is developed based on five main contexts: geography, function, form, society, and culture (Danilova and Bakshutova, 2020). Local identity can influence citizens’ support for local behaviors, both personally and socially (Belanche et al., 2017). In fact, a culture-led urban renewal that leverages historical assets and local culture is the direction of contemporary urban renewal. Urban residents take pride in their urban identity, attracting tourists from other places who seek to experience and implement the unique characteristics of the destination. Targeted urban renewal driven by a sense of place and cultural identity can be sustainable (Hwang, 2014).

Examples have demonstrated that after building revitalization, there is an increase in business opportunities for entertainment, attracting non-local visitors who generate economic activities (Simard and Mercier, 2001; Elsorady, 2014). Value enhancement is not limited to a quantitative economic approach; it also understands the historical background of the building and encompasses the connection with local identity. The contribution of building of revitalization can create a sense of place which can relate to collective implication. Carr et al. (2016) confirmed the social cohesion, community identity, or affiliation that social groups derive from specific heritage contributes to this value enhancement. Yung and Chan (2012) argue that local sustainability behavior is influenced by built heritage projects, where locals can enhance their sense of cultural identity through the atmosphere of the place and the quality of the environment (Jackson, 2008). Locals play a crucial role as a motivational force behind the design for the conservation of built heritage, establishing a link to community cultural identity (Tweed and Sutherland, 2007).

Based on this, the following hypothesis is proposed:

Hypothesis 1. Heritage building revitalization is positively related to local cultural identity.

### 2.4 Relationship between cultural identity and place attachment through tourism

Hidalgo and Hernandez (2001) highlight that culture and place identity refers to home, neighborhood and city have positive relationship. The U-shaped relationship between the scale of place and the strength of place attachment may lead visitors to prioritize visiting a city rather than a neighborhood (Bernardo and Palma-Oliveira, 2013). McIlvenny et al. (2009) demonstrate that place attachment and identity play important roles in people’s social and psychological empowerment. They influence each other and jointly determine social and behavioral decisions. Although different cultural backgrounds can create attachment to the same place, multi-racial integration maintains identification within the country (Ujang and Zakariya, 2015). Belanche et al. (2021) which highlighted different approach of place identity is the self that define the individuals identity in relation to the physical environment by means of conscious ideas, belief, preference, feeling, values, goals, and behavioral tendencies and skills relevant to the environment. Individual involves self-awareness of one of the member in their group (Tajfel and Turner, 1979). Therefore, the present group which create self –awareness as a root of culture identity because of psychological to special location or environment, this approach will consider place identity as a feature of the place based on group collective attribution from individual place attachment because of self –identification. Based on this relation, the following hypothesis is proposed:

Hypothesis 2. Local cultural identity is positively related to place attachment.

### 2.5 Effect of place attachment on visitors’ post-experience behavior

The concept of post-experience behavior was initially proposed by Dong and Siu (2013) to describe the intensification and extension of behavioral tendencies among theme park visitors during and after their play experiences. This concept has also been applied to describe visitors’ behavioral tendencies after nature tourism experiences (Sthapit et al., 2022). Recent research suggests that place attachment is an important prerequisite for the emergence of post-experience behavior (Kim and Lee, 2022).

Post-experience behavior can be divided into two dimensions: experience intensification and experience extension. Experience intensification occurs when visitors who have experienced historic district buildings show a strong willingness to make purchases and feel attached to the place (Alawadi, 2017; Li et al., 2021). Similarly, taking commemorative photographs is a common choice for visitors who have a sense of attachment to the location of historic buildings (Gregory, 2015). These behaviors are examples of experience intensification, as they represent tourists reinforcing their own experiences in the present context.

Experience extension, the other dimension of post-experience behavior, involves sharing the positive aspects of one’s experience with others (Arnould and Price, 1993). Visitors’ intention to revisit a destination is influenced by the meaning locals hold for that destination (Zhang et al., 2018). People with a stronger sense of place attachment are more likely to revisit a particular destination and expand their experiences. Similarly, if a local resident has a strong sense of place attachment, they are more likely to provide information about local excursions when asked (Kim and Lee, 2022). These behaviors are important components of experience extension (Dong and Siu, 2013). Therefore, based on the literature highlighting the impact of place attachment on both dimensions, the following hypotheses are proposed:

Hypothesis 3a. Place attachment is positively related to experience intensification.

Hypothesis 3b. Place attachment is positively related to experience extension.

### 2.6 Knowledge transfer as a moderator in the relationship between the effectiveness of heritage building revitalization and local cultural identity

Knowledge transfer is defined by Alavi and Leidner (2001) as the process of learning from the knowledge of one organization to another to maintain or gain a competitive advantage. Knowledge transfer can have significant socio-economic impact, particularly on innovation management (Grosse Kathoefer and Leker, 2012). In the context of tourism, effective knowledge transfer can contribute to the competitiveness of tourism destinations and products (Baggioa and Cooperb, 2010). Knowledge transfer is a key element of tourism innovation (Makkonen et al., 2018). It should be maximized to promote tourism development (Werner et al., 2015). Under this logic, knowledge transfer can stimulate the effectiveness of heritage building in revitalization. Facilitating more innovative product for the visitors.

Ashforth (2001) identifies the critical motives in knowledge transfer processes, including the elements of identity, sense-making, self-control and sense of belonging, concluding that such motives stimulate the socialization process. Knowledge transfer can shape individual behavior (Masrek et al., 2011). Kane et al. (2005) highlighted knowledge transfer which have relationship to the group and social identity.

The greater frequency of knowledge transfer in a group which had significant consequences for the performance of those group, the more the understanding toward the identity of the place and physical environment. This helps strengthen individuals’ level of engagement and attachment to the place. Consequently, they are more willing to engage with the social activities and connect to the social and psychical aspects of the place (Seamon, 1979). The engagement of the social and physical environment, social beings never escape their embeddedness (Harvey, 1996). Therefore, under this logic, knowledge transfer may affect culture identity for individuals.

Revitalization of the ancient city of Chiang Mai, for example, has created a cultural identity among locals based on the uniqueness of their city (Phetsuriya and Heath, 2021). Tan and Tan (2020) point out that even locals may not fully realize the uniqueness of their own city and require effective knowledge transfer to gain a deeper understanding. Neglecting or misusing urban heritage can lead to the loss of common urban memory among the younger generation (Cittati et al., 2022). Therefore, the revitalization and proper promotion of heritage buildings can ensure the transfer of common urban memory to the next generation through knowledge, stimulating their sense of cultural identity. Based on this, the following hypothesis is proposed:

Hypothesis 4. Knowledge transfer moderates the effect of heritage building revitalization on local cultural identity, with a stronger effect when knowledge transfer is present.

### 2.7 Cultural activities engagement as a moderator in the relationship between local cultural identity and place attachment

Activities engagement is defined by Vivek et al. (2014) as the cognitive and emotional absorption that occurs during an activity. Positive emotions are used in a wide range of studies to explain activity engagement (Mageau and Vallerand, 2007). Cultural activities engagement in relationship with local identity and place attachment.

According to social identity theory, individuals are motivated to participate in activities associated with their own group (“us”) rather than activities associated with other groups (“them”) (Tajfel et al., 1979). Attachment theory suggests that people can develop attachments to both individuals and objects (Giuliani, 2003). Therefore, individuals with a strong cultural identity may develop a sense of attachment to a place through cultural activities that reflect their own culture. Schilar and Keskitalo (2018) argue that active engagement in activities is an outward expression of place attachment, even if the cultural identities of those involved in the activities are not homogeneous. On the other hand, a higher level of cultural or ethnic identity is required for the development of place attachment in highly engaged cultural activities. For example, Turkish immigrants living in Germany reinforce their cultural identity differences from the locals through cultural activities in mosques. These highly engaged cultural activities strengthen Turkish cultural identity and attachment to their homeland (Ehrkamp, 2005; Demmrich and Arakon, 2021). Research by Freeman et al. (2022) suggests that cultural background identity strongly influences cultural activities engagement and place attachment.

Hypothesis 5. Cultural activities engagement moderates the effect of local cultural identity on place attachment in that the effect is stronger for cultural activities engagement.

### 2.8 Effect of emotional attachment on place attachment

Emotional attachment is defined as an emotionally charged connection between a person and a particular object (Thomson et al., 2005). A stronger attachment to a specific target induces an emotionally charged state of mental readiness that influences the individual’s emotions, cognition and behavior toward the object (Hang et al., 2020). When emotional attachment becomes strong, it manifests as external expressions related to love and passion (Ladhari et al., 2020). Initially, emotional attachment was thought to be associated with negative emotions, but research also suggests the involvement of positive emotions in the construction of emotional attachment (Goodall, 2015). Furthermore, Hosany et al. (2017) found that both positive and negative affect are important antecedents of place attachment, as evidenced by proximity-seeking behavior, separation pain, feelings of security provided by attachment objects and mourning for loss (Sperling and Berman, 1994).

Trauer and Ryan (2005) argue that place attachment and emotional attachment are strongly related. Place memory and place expectancy are considered important predictors of place attachment by Chen et al. (2014), and place expectancy and emotional attachment influence each other. Bora and Voiculescu (2021) suggest that the more negative a person’s memory of a place is, the more challenging it becomes for emotional attachment to develop, resulting in weaker attachment to that place. Loureiro (2014) argues that positive emotions lead to stronger place attachment.

Hypothesis 6. Emotional attachment is positively related to place attachment.

**FIGURE 5:**
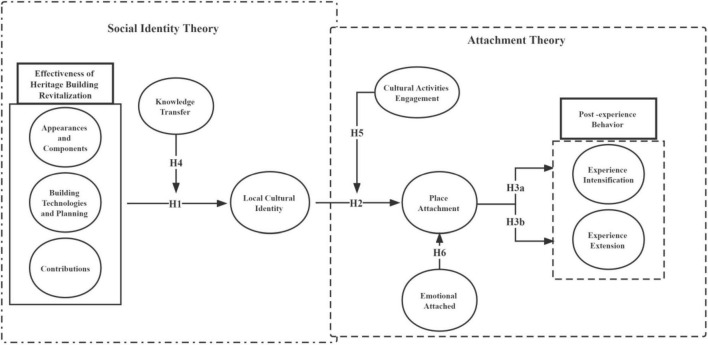
Conceptual model of this article.

Therefore, the following conceptual model as shown in Figure 5 is proposed.

## 3 Methodology

### 3.1 Methods selection

The nature of the research determines the methodology (Lee et al., 2016). In order to explore the relationship between the models in the conceptual model, qualitative research is not appropriate. Instead, Structural Equation Modeling (SEM) in quantitative research is suitable for exposing the complex relationships between the constructs in the above model (Mikulić and Ryan, 2018). In addition, for the measurement of the constructs, the questionnaire method is currently widely adopted. By filling out the questionnaire, the respondents’ mental state can be fully recorded by the data (Mikulić and Ryan, 2018). Therefore, collecting data through questionnaire method and modeling the data within the framework of SEM was selected as the methodological framework for this study. Because of the second-order modeling involved in this study, as well as the fact that this paper was conducted in an exploratory research framework. Therefore covariance-based structural equation modeling (CB-SEM) and partial least squares structural equation modeling (PLS-SEM), the latter was selected as the data processing method for this study (Dash and Paul, 2021).

### 3.2 Measurement

The measurement items used in this study were derived from well-established scales in the historical literature. The measurement of Effectiveness of Heritage Building Revitalization referred to the scale of Chang et al. (2020), which consists of 3 dimensions: Appearances and Components, Building Technologies and Planning and Contributions. The measurement of Local Cultural Identity is based on the scale of Zeng and Xu (2021), and the measurement of Knowledge Transfer is based on the scale of Liu (2018). Cultural Activities Engagement refers to the scale of Peng et al. (2023). Place Attachment refers to the scale of Von Wirth et al. (2016). Emotional Attached refers to the scale of Kowalczyk and Pounders (2016). Post Experience Behavior was divided into two dimensions, Experience Intensification and Experience Extension. These two dimensions were measured with reference to the scales of Dong and Siu (2013) and Kim and Lee (2022). Therefore, a total of 44 questions were included in this study to measure all constructs. The specific construct measurement items and their sources were presented in Table 1.

**TABLE 1:** Construct measurement items and source.

Construct	Items	
**Effectiveness of Heritage Building Revitalization**		
Appearances and Components (Dimension)	The building is generally well preserved.	Chang et al., 2020
	Cultural elements inside the revitalized building have been preserved (e.g., paintings).	
	There are representative elements of the historic building that have been preserved and displayed.	
	New artistic elements have been added to the architectural elements (e.g., wall paintings).	
	The facades of the revitalized buildings do not have the feeling of being in disrepair.	
	The signage of the building before and after the revitalization has been kept consistent.	
Building Technologies and Planning (Dimension)	The interior of the revitalized building is fitted with a large amount of glass (e.g., glass canopy).	Chang et al., 2020
	New technical means of presentation (e.g., 3D virtual technology) are introduced to the interior of the revitalized building.	
	New functional areas are introduced inside the revitalized building.	
	The walls of the revitalized building are solid.	
Contributions (Dimension)	The stores in the revitalized building are full of local cultural products, while the food in the restaurant is full of local specialties.	Chang et al., 2020
	Local designers can use the stores in the revitalized building to showcase their products.	
	The revitalized building is a powerful way to promote local culture.	
	I can learn about the history of the building in the revitalized building.	
Local Cultural Identity	I feel good about myself as a local resident.	Zeng and Xu, 2021
	I have a sense of satisfaction as a local resident.	
	I have a sense of accomplishment as a local resident.	
	I am proud to be a local resident.	
Knowledge Transfer	An introduction to historical knowledge can strengthen my understanding of revitalized architecture.	Liu, 2018
	The introduction to historical knowledge will accelerate my understanding of revitalized architecture.	
	The presentation of historical knowledge stimulates my interest in understanding the history of revitalized architecture.	
	The results of the knowledge transfer of the history of the revitalized building are satisfactory.	
Cultural Activities Engagement	I had a feeling of self-liberation during cultural experience of the revitalized buildings.	Peng et al., 2023
	I had a feeling of refreshment during cultural experience of the revitalized buildings.	
	I had a sense of freedom during cultural experience of the revitalized building.	
	I feel completely immersed in this cultural experience of the revitalized building.	
	I experience a feeling of being one with my surroundings during cultural experience of the revitalized building.	
Place Attachment	The city where this historical building locates is not the ideal place for me to live.	Von Wirth et al., 2016
	I would live in another place other than the city where this historical building locates.	
	I don’t have anything in common with the city where this historical building locates.	
	I don’t identify with the people who live in this city.	
	I don’t feel integrated in the city where this historic building locates.	
Emotional Attached	I am emotionally connected with the city where this historical building locates.	Kowalczyk and Pounders, 2016
	The city where this historical building locates says something true and deep about who lam as a person.	
	If the city where this historical building locates was to no longer be in the spotlight, I would feel anxiety.	
Experience Intensification	I would buy souvenirs in this revitalized buildings.	Kim and Lee, 2022
	I take memorable photos inside this revitalized buildings.	
	The photos related to this revitalized building make my visit more impressive.	
Experience Extension	I will visit this revitalized building again.	Kim and Lee, 2022
	I would recommend this revitalized building to my relatives and friends.	
	I often recall my experience in this revitalized building.	
	When I get a chance, I recommend this building to people who ask me for advice.	Dong and Siu, 2013
	I will share this experience with my family and friends.	
	After visiting the architecture, my vision and knowledge have been expanded.	

The questionnaire was prepared in both English and Chinese as it was distributed in Hong Kong. Back-translation was adopted to enhance the survey’s credibility (Ozolins et al., 2020). The study aimed to examine the psychological behavior of visitors toward revitalized historic buildings, so the authors sought assistance from three industry experts in the field of heritage building revitalization and tourism. After their translation and double-checking, the final version of the questionnaire was prepared.

The researcher conducted a pre-test of the questionnaire on a 50-person size at the target sampling site prior to the formal distribution of the questionnaire (Perneger et al., 2015). The purpose to enhance the readability and reliability of the questionnaire. According to the principle of deleting questions with factor loading below 0.7, we deleted questions that could not explain the concepts adequately. In this way, the questionnaire was purified. Finally, AC4, AC5, and AC6 in Appearances and Components were deleted, and BTP1 in Building Technologies and Planning was deleted. CAE1 and CAE3 in Cultural Activities Engagement were deleted. EI3 in Experience Intensification and EE3 in Experience Extension were deleted. The final questionnaire therefore had a cumulative total of 35 questions on the construct measure.

### 3.3 City selection and sample collection

Owing to the distinctive achievements of Hong Kong in historic building revitalization, this study focused on collecting samples from the most popular locations in Hong Kong.

The questionnaire consisted of three parts. The first part comprised a screening question to confirm whether the respondents were visitors who had visited Tai Kwun, Police Married Quarters, Mei Ho House, or The Mills. These four buildings are highly popular in Hong Kong and serve as the source buildings for the development of the effectiveness scale of heritage building revitalization (Lam et al., 2022). The second part consisted of 44 questions on ten research model constructs. Respondents were asked to recall their psychological feelings during their visit to the aforementioned revitalized buildings and indicate their level of agreement on a Likert scale. A response of “1” indicated strong disagreement, whereas “7” indicated strong agreement. To mitigate the possibility of common method bias, the constructs in the questionnaire were presented in a random order rather than following the consistent arrangement of Table 1. Additionally, the five measurement items of Place Attachment were presented using negative questioning instead of the original positive questioning format (Chang et al., 2020). The final part of the questionnaire involved collecting personal information.

The questionnaire was distributed among local people, through the researchers’ personal connections and on-site distribution at the exits of the four revitalized architectural sites (Tai Kwun, Police Married Quarters, Mei Ho House, or The Mills).

The reason why we selected the four sites in Hong Kong since the cities develops rapidly, local government allowed a rich and distinct culture to evolve which can reflect by the architecture. Many old buildings found in Hong Kong which provide a strong culture characteristic (Lam et al., 2022). Furthermore, the flagship heritage building in Hong Kong with economic value to the local community. The selection of Tai Kwun, Police Married Quarters, Mei Ho House and Mills since they are holding a high level of symbolic important includes for historical, political and economic centrality (Barber, 2020). We decided to collect data from those places since we can verify the right respondents and the result can create credibility.

To ensure the sampled subjects were representative of the overall visitor sample, the field questionnaire recovery time spanned weekdays and weekends. Only seven out of 359 respondents indicated that they had not visited the revitalized buildings mentioned above, even though they were local Hong Kong residents. Therefore, the final number of valid questionnaires was 348.

### 3.4 Data analysis method

Several analysis methods exist for examining psychological latent variables. However, given that the theoretical model in this paper incorporates mediating and moderating variables, as well as multiple dependent variables, structural equation modeling (SEM) is deemed more suitable for uncovering the relationships between the respective variables (Duncan, 2014). There are several platforms for running the PLS-SEM algorithm, but the most widely accepted is smartPLS (Dash and Paul, 2021). Thus, the data analysis platform selected for this study is PLS-SEM using smartPLS 4.0.9.2.

## 4 Findings

### 4.1 Demographic information

Table 2 presents the demographic information of the respondents who completed the valid questionnaire. The gender distribution was predominantly female (60.06%). The age range with the highest number of respondents was 18–24 years old, and as age increased, the number of respondents decreased. The proportion of respondents with and without a local place of birth was similar. Lastly, the largest occupation group among the respondents was students (55.17%), followed by enterprise employees (31.03%).

**TABLE 2:** Demographic information.

Gender	Frequency	Percent	City of birth is Hong Kong	Frequency	Percent
Male	139	39.94%	Yes	161	46.26%
Female	209	60.06%	No	187	53.74%
Age			Occupation		
18–24	232	66.67%	Students	192	55.17%
25–34	92	26.44%	Enterprise employee	108	31.03%
35–44	10	2.87%	Civil service	3	0.86%
45–54	8	2.30%	Freelance work	36	10.34%
55–64	1	0.29%	Out of retirement	3	0.86%
>65	5	1.44%	Others	6	1.72%

### 4.2 Construct reliability and validity

Table 3 displays the mean, standard deviation and factor loading of the measurement items for all constructs. According to Hair et al. (2019), items with factor loadings below 0.7 should be removed. Therefore, not all 44 questions were used in the final analysis, and a total of nine items were removed. Additionally, the higher-order construct Effectiveness of Heritage Building Revitalization was measured using the repeated indicator method. To ensure consistency in the interpretation of lower-order constructs, one additional question (AC5) was removed, resulting in a consistent number of questions across the three dimensions.

**TABLE 3:** Descriptive statistics and factor loading of items.

	Mean	Standard deviation	Factor loading	Excess kurtosis	Skewness
AC1	5.640	1.015	0.909	−0.927	−0.204
AC2	5.514	1.022	0.826	−0.276	−0.273
AC3	5.560	0.951	0.816	0.235	−0.564
AC4	5.206	1.351	0.643	−1.032	−0.260
AC5	5.329	1.317	0.749	−0.366	−0.465
AC6	5.103	1.174	0.616	−0.509	−0.180
CT1	4.951	1.233	0.794	−0.555	−0.146
CT2	5.540	1.102	0.646	0.995	−0.726
CT3	5.809	0.862	0.811	−0.255	−0.345
CT4	5.511	1.128	0.834	1.185	−0.922
BTP1	4.580	1.484	0.331	−0.267	−0.359
BTP2	5.246	1.208	0.860	−0.802	−0.227
BTP3	5.403	1.267	0.812	0.152	−0.740
BTP4	5.823	1.024	0.759	−0.897	−0.425
KT1	5.446	1.096	0.921	−0.700	−0.273
KT2	5.589	1.070	0.893	−0.532	−0.471
KT3	5.677	1.106	0.920	−0.468	−0.568
KT4	5.423	1.052	0.868	−0.682	−0.098
LCI1	5.354	1.017	0.856	−1.051	0.228
LCI2	5.520	0.830	0.911	−0.407	0.041
LCI3	5.583	0.990	0.884	−0.704	−0.187
LCI4	5.494	0.999	0.765	−0.227	−0.252
CAE1	5.477	1.304	0.697	4.274	−1.750
CAE2	5.340	1.012	0.727	−0.086	−0.356
CAE3	5.409	0.948	0.553	−0.553	0.113
CAE4	5.283	0.918	0.728	−0.258	0.055
CAE5	5.049	1.050	0.872	−0.644	0.215
PA1	4.769	1.665	0.916	−0.253	−0.908
PA2	4.469	1.745	0.905	−0.811	−0.474
PA3	4.689	1.839	0.911	−1.097	−0.325
PA4	5.011	1.582	0.915	−0.385	−0.702
PA5	4.989	1.611	0.889	−0.317	−0.793
EI1	4.234	1.368	0.976	−0.394	0.291
EI2	4.830	1.479	0.813	−0.642	−0.354
EI3	5.594	1.200	0.175	−0.623	−0.442
EA1	4.917	1.439	0.864	−1.007	−0.357
EA2	5.391	1.089	0.890	0.202	−0.479
EA3	5.074	1.217	0.876	1.231	−0.659
EE1	5.489	1.121	0.786	−0.894	−0.253
EE2	5.469	1.158	0.860	−0.495	−0.428
EE3	4.891	1.267	0.482	−0.746	−0.074
EE4	5.449	1.072	0.922	−0.508	−0.452
EE5	5.549	1.024	0.736	−0.966	−0.212
EE6	5.637	1.010	0.854	0.489	−0.679

Table 4 presents the Cronbach’s alpha values, composite reliability (CR) and average variance extracted (AVE) for each construct. Based on Hair et al. (2019), values above 0.7 for Cronbach’s alpha and CR, as well as AVE values above 0.5, demonstrate the reliability and convergent validity of the data. It is worth noting that Experience Intensification’s composite reliability (rho_a) exceeds 1. This is not an unusual result, but there is no literature yet highlighting that this value exceeds 1 as a sign of serious problems with the data. Therefore, the authors believe that this value does not yet validate that there is a problem with composite reliability.

**TABLE 4:** Reliability and convergent validity test.

	Cronbach’s alpha	CR	AVE
Appearances and Components	0.872	0.872	0.797
Building Technologies and Planning	0.748	0.764	0.664
Contributions	0.776	0.778	0.692
Cultural Activities Engagement	0.791	0.880	0.704
Effectiveness of Heritage Building Revitalization	0.891	0.899	0.540
Emotional Attached	0.853	0.884	0.769
Experience Extension	0.900	0.882	0.699
Experience Intensification	0.808	1.101	0.825
Knowledge Transfer	0.923	0.941	0.812
Local Cultural Identity	0.876	0.878	0.732
Place Attachment	0.947	0.955	0.823

Table 5 shows the results of the discriminant validity test. Following the criteria of Fornell and Larcker (1981) and Ab Hamid et al. (2017), as the square root of AVE is greater than the correlation coefficient between the corresponding constructs, all constructs in this study are independent of one another, indicating sufficient discriminant validity for the model.

**TABLE 5:** Discriminant validity test.

	Appearances and components	Building technologies and planning	Contributions	Cultural activities engagement	Emotional attached	Experience extension	Experience intensification	Knowledge transfer	Local cultural identity	Place attachment
Appearances and components	**0.893**									
Building technologies and planning	0.563	**0.815**								
Contributions	0.756	0.55	**0.832**							
Cultural activities engagement	0.429	0.546	0.432	**0.839**						
Emotional attached	0.174	0.488	0.249	0.533	**0.877**					
Experience extension	0.577	0.688	0.48	0.534	0.717	**0.836**				
Experience intensification	0.35	0.296	0.282	0.437	0.372	0.403	**0.908**			
Knowledge transfer	0.628	0.536	0.572	0.417	0.239	0.544	0.428	**0.901**		
Local cultural identity	0.546	0.525	0.53	0.587	0.526	0.618	0.327	0.503	**0.856**	
Place attachment	0.212	0.237	0.09	−0.128	0.111	0.278	−0.375	0.032	0.145	**0.907**

The value on the diagonal is the square root of the average variance extracted (AVE) value.

### 4.3 Results of PLS analysis

#### 4.3.1 Direct effect analysis

According to Hair et al. (2012), a total of 352 valid samples and 5000 subsamples were analyzed to assess the significance of the paths.

Table 6 displays the analysis of the direct effects between the constructs. Appearances and Components, Building Technologies and Planning and Contributions are all found to be valid indicators of Effectiveness of Heritage Building Revitalization, confirming the findings of Lam et al. (2022). Furthermore, Appearances and Components (β_Appearances and Components –__>__Effectiveness of Heritage Building Revitalization_ = 0.450, *p* < 0.001) and Contributions (β_Contributions –>_ _Effectiveness of Heritage Building_ _*Revitali*zation_ = 0.325, *p* < 0.001) are explored that contribute most to Effectiveness of Heritage Building Revitalization, rather than Building Technologies and Planning (β_Building Technologies_ _*and Planning –* >_ _Effectiveness of Heritage Building Revitalization_ = 0.374, *p* < 0.001).

**TABLE 6:** PLS direct effect.

Hypothesis	Path	Coefficients	T statistics	Hypothesis result
	Appearances and Components - > Effectiveness of Heritage Building Revitalization	0.450***	29.938	
	Building Technologies and Planning - > Effectiveness of Heritage Building Revitalization	0.325***	19.272	
	Contributions - > Effectiveness of Heritage Building Revitalization	0.374***	27.993	
H1	Effectiveness of Heritage Building Revitalization - > Local Cultural Identity	0.549***	12.640	Support
H2	Local Cultural Identity - > Place Attachment	0.285***	4.887	Support
H3a	Place Attachment - > Experience Intensification	−0.375***	7.211	Support
H3b	Place Attachment - > Experience Extension	0.278***	7.076	Support
H6	Emotional Attached- > Place Attachment	0.168*	2.194	Support

***p < 0.001, *p < 0.05.

H1 and H2 are supported because Effectiveness of Heritage Building Revitalization is shown to have a positive effect on Local Cultural Identity (β_Effectiveness of Heritage_ _*Building Revitali*zation – >_ _Local Cultural Identity_ = 0.549, *p* < 0.001). In addition, Local Cultural Identity promotes Place Attachment (β_Local Cultural Identity – >_ _Place Attachment_ = 0.285, *p* < 0.001).

Interestingly, the results indicate that Place Attachment has a negative effect on Experience Intensification (β_Place Attachment__ – >__Experience Intensification_ = −0.375, *p* < 0.001) and positive effect on Experience Extension (β_Place_ _*Attachment –* >_ _Experience Extension_ = 0.278, *p* < 0.001). Ding and Hung (2021) suggest that Experience Extension is associated with individuals’ willingness to recommend, so people with a high level of Place Attachment readily mention the positive aspects of the building to others. However, local visitors are already familiar with the historical background of these buildings and may not make frequent visits. Therefore, Place Attachment is negatively related to Experience Intensification, whereas Experience Extension demonstrates a positive approach. Consequently, H3a is rejected, but H3b is supported. The moderating effect hypotheses, H4 and H5, will be further discussed in the next section.

H6 is also supported because Emotional Attached to revitalization by locals significantly leads to Place Attachment (β _Emotional Attached – >_ _Place Attachment_ = 0.168, *p* < 0.05).

#### 4.3.2 Indirect effect analysis

The results presented in Table 7 represent the analysis of the mediating effects. Consistent with the previous discussion, Effectiveness of Heritage Building Revitalization positively influences residents’ Experience Extension and negatively influences Experience Intensification through mediating variables.

**TABLE 7:** PLS mediating effect test result.

Path	Coefficient	T statistics	*P*-values
Effectiveness of Heritage Building Revitalization - > Local Cultural Identity - > Place Attachment - > Experience Intensification	−0.059	3.965	0.000
Effectiveness of Heritage Building Revitalization - > Local Cultural Identity - > Place Attachment - > Experience Extension	0.044	3.951	0.000
Emotional Attached - > Place Attachment - > Experience Extension	0.047	2.001	0.045
Emotional Attached - > Place Attachment - > Experience Intensification	−0.063	2.066	0.039

**FIGURE 6:**
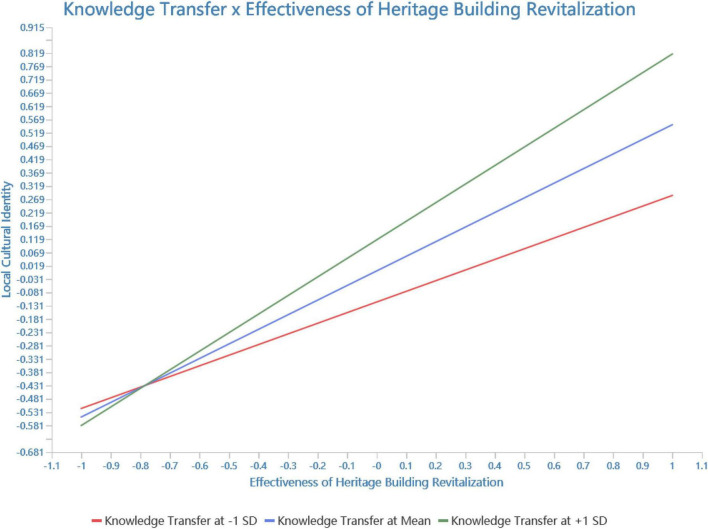
Simple slope analysis.

The results of the test for moderating effect are presented in Table 8. The moderating effect of Knowledge Transfer is demonstrated (β = 0.149, 95% CI = [0.070, 0.223]). This implies that the positive effect of Heritage Building Revitalization on Local Cultural Identity is enhanced with the enhancement of Knowledge Transfer. Hence, H4 is supported. This result aligns with Cheung and Chan (2014), suggesting that managers of as all sized buildings can consider strengthening the construction of guided tours, which, in turn, will enhance Knowledge Transfer. Figure 6 presents a simple slope analysis of Effectiveness of Heritage Building Revitalization moderated by Knowledge Transfer. The effect of the interaction term on local cultural identity significantly differs when adding the mean or subtracting one standard deviation.

**TABLE 8:** PLS moderating effect test result.

Hypothesis	Path	Coefficient	95% CI LL	Hypothesis result
H4	Knowledge Transfer x Effectiveness of Heritage Building Revitalization - > Local Cultural Identity	0.149	0.070| 0.223	Support
H5	Cultural Activities Engagement x Local Cultural Identity - > Place Attachment	−0.019	−0.134| 0.104	Reject

**FIGURE 7:**
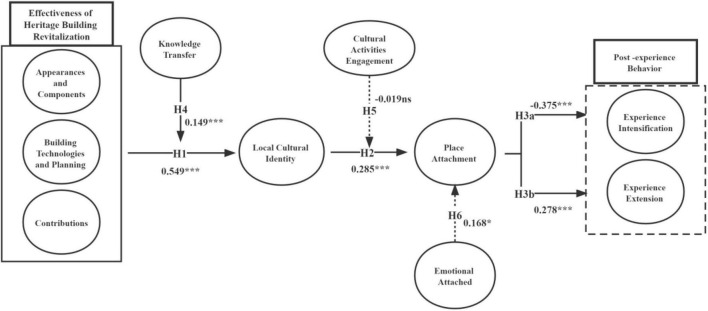
Test result of conceptual model. ***p < 0.001, *p < 0.05, ns *p* < 0.05.

However, the moderating effect of Cultural Activities Engagement is not demonstrated in the relationship between Local Cultural Identity and Place Attachment (β = −0.019, 95% CI = [0.134, 0.104]). This result indicates that H5 is rejected. Thus, the effect of Local Cultural Identity may not be affected by cultural activities engagement as a moderator in relation to Place Attachment. Figure 7 represents the final test result of the conceptual model.

Table 9 presents the R square and Q square values of all dependent variables for both models. Following the criteria of Cohen (1988) and Rehman Khan and Yu (2021), as all R square values exceed the minimum threshold of 0.02 and all Q square values are greater than 0, the variance of the exogenous variable in the model can well explain the variance of the endogenous variable. Additionally, the predictive relevance of the model is deemed satisfactory.

**TABLE 9:** R square and Q square of the dependent variable.

Endogenous variable	R square	Q square
Effectiveness of Heritage Building Revitalization	1.000	0.535
Experience Extension	0.077	0.038
Experience Intensification	0.141	0.101
Local Cultural Identity	0.412	0.289
Place Attachment	0.109	0.078

## 5 Conclusion

Findings confirms the positive impact of heritage building revitalization on visitors’ post experience behavior. Firstly, results confirm that heritage building revitalization positively supports local visitors’ cultural identity and place attachment. The effectiveness of heritage building revitalization lies on appearances and components, technology as well as planning and contributions. Findings postulated that all these three elements are positively related to visitors’ cultural identity and emotion. These help facilitate place attachment (Morgan, 2010; Hosany et al., 2017).

Secondly, place attachment is negatively related to experience intensification. Conversely, place attachment is positively related to experience extension. Findings confirmed that emotional attachment influence the level of place attachment. Emotional attachment also demonstrates a positive relationship with place attachment. The findings are somewhat consistent with the literature findings Positive emotions in tourism favor a valence-based approach (Russell, 1980) and research has shown that positive emotions toward a place reinforce a sense of attachment (Morgan, 2010).

Thirdly, knowledge transfer is positively related to heritage building revitalization. It can be explained by the fact that knowledge transfer facilitates visitors’ understanding about the building such as building’s history and other factual information. This help evokes a sense of intense vicariousness and lead to place attachment. The finding is consistent with the previous literature findings that heritage buildings hold a strong position in educating visitors and creating a sense of place (Hou and Wu, 2020).

Lastly, cultural activities engagement is negatively related to the relationship between local cultural identity and place attachment. This implies that cultural activities engagement does not enhance the effect of local cultural identity on place attachment (Rajadurai et al., 2023). It can be explained by the fact that local visitors, who are already familiar with the historical buildings and the community, may not engage in purchase behavior on-site. Nor do they make efforts to make their experience a tangible one.

### 5.1 Implications of the study

From a managerial perspective, positive results of heritage building revitalization provide insights for destination marketers. The appearance components, technologies and planning and contributions contribute to the identification and self-belongingness of the place. Developers involved in the planning of heritage building revitalization need to consider the benefits not only limited to the building itself and the surrounding environment but also the marketing of conservation strategies and the facilitation of service provision and delivery.

By showcasing tangible historic buildings whilst providing value to locals and fulfilling the role of cultural inheritance, marketers can adopt knowledge transfer to enhance visitors’ positive psychological wellbeing. Knowledge transfer delivers additional information to visitors, helping them better understand the history or background of the place. Marketers can craft stories and deliver messages through heritage building revitalization, enabling visitors to comprehend the importance of long-term building preservation for sustainability.

Additionally, the findings indicate that emotional attachment leads to satisfaction and cultural identity. Positive emotions resulting from service encounters and cultural experiences may lead to positive word-of-mouth recommendations. Place attachment creates memories that reinforce visitor emotions associated with a place (Grisaffe and Nguyen, 2011). Destination management should aim to evoke positive emotions and foster place attachment through knowledge transfer to visitors.

Furthermore, increasing place identity can be achieved by providing more cultural explanations or activities. Adopting more technologies to enhance the visitor experience can improve their intention to revisit.

From an academic perspective, study expands upon existing research by addressing the viewpoint of visitors and measuring post-experience behavior related to heritage building revitalization. It establishes the roles of heritage building revitalization and initiates a conceptual framework for further evaluation. The integration of social identity theory and attachment theory in a cross-disciplinary manner highlights the importance of heritage building revitalization in relation to visitors’ cultural identity and place attachment, rather than solely focusing on urban development and planning. Future studies can explore visitors’ psychological perspectives to better understand their post-experience behavior.

### 5.2 Limitations and future study

The findings of this study are subject to certain limitations. Firstly, the sampling was limited to local visitors, and the results may differ for overseas visitors. Local visitors, being familiar with the background of the heritage buildings, may have different perspectives compared within international visitors when visiting these heritage sites. Secondly, the city selection was limited to Hong Kong’s heritage buildings, and different results may be obtained in cross-pacific countries due to different policies, planning and management as well as varying rules and regulations.

From a destination management perspective, future studies can examine the frequency of visits to these attractions by distinguishing between first-time visitors and repeat visitors. Capturing visitors’ pre- and post-visiting experiences to heritage buildings can provide further insights into their visiting intentions. To minimize bias, a mixed methods approach can be adopted, combining qualitative and quantitative research methods to gain a comprehensive understanding of visitors’ needs and expectations regarding the services provided by heritage buildings.

## Data availability statement

The raw data supporting the conclusions of this article will be made available by the authors, without undue reservation.

## Ethics statement

Ethical review and approval was not required for the study on human participants in accordance with the local legislation and institutional requirements. Written informed consent from the patients/participants or patients/participants legal guardian/next of kin was not required to participate in this study in accordance with the national legislation and the institutional requirements.

## Author contributions

SC: Writing—original draft, Writing—review and editing. WL: Writing—original draft, Writing—review and editing. BT: Data curation, Methodology, Writing—original draft, Writing—review and editing. ZC: Writing—original draft.
